# Supplementary material for: Host origin of microbiota drives functional recovery and Clostridioides difficile clearance in mice
Source: mBio. 2025 Jun 2;16(7):e01108-25. doi: 10.1128/mbio.01108-25 (PMC12239565; doi:10.1128/mbio.01108-25)
Supplement: Supplemental Figures — Figures S1 to S11 and supplemental methods. [file mbio.01108-25-s0001.pdf]

Supplementary Figures and Methods for

Host origin of microbiota drives functional recovery and *Clostridioides difficile* clearance in mice

Authors: Sophie A. Millard[a], Kimberly C. Vendrov[b,c], Vincent B. Young[b,c], Anna M. Seekatz[a*]

[a]*Department of Biological Sciences, Clemson University, Clemson, SC 29634, USA*

[b]*Department of Internal Medicine, Division of Infectious Disease, University of Michigan, Ann Arbor, MI 48109, USA*

[c]*Department of Microbiology and Immunology, University of Michigan, Ann Arbor, MI 48109, USA*

[*]Corresponding author

Supplemental Figures

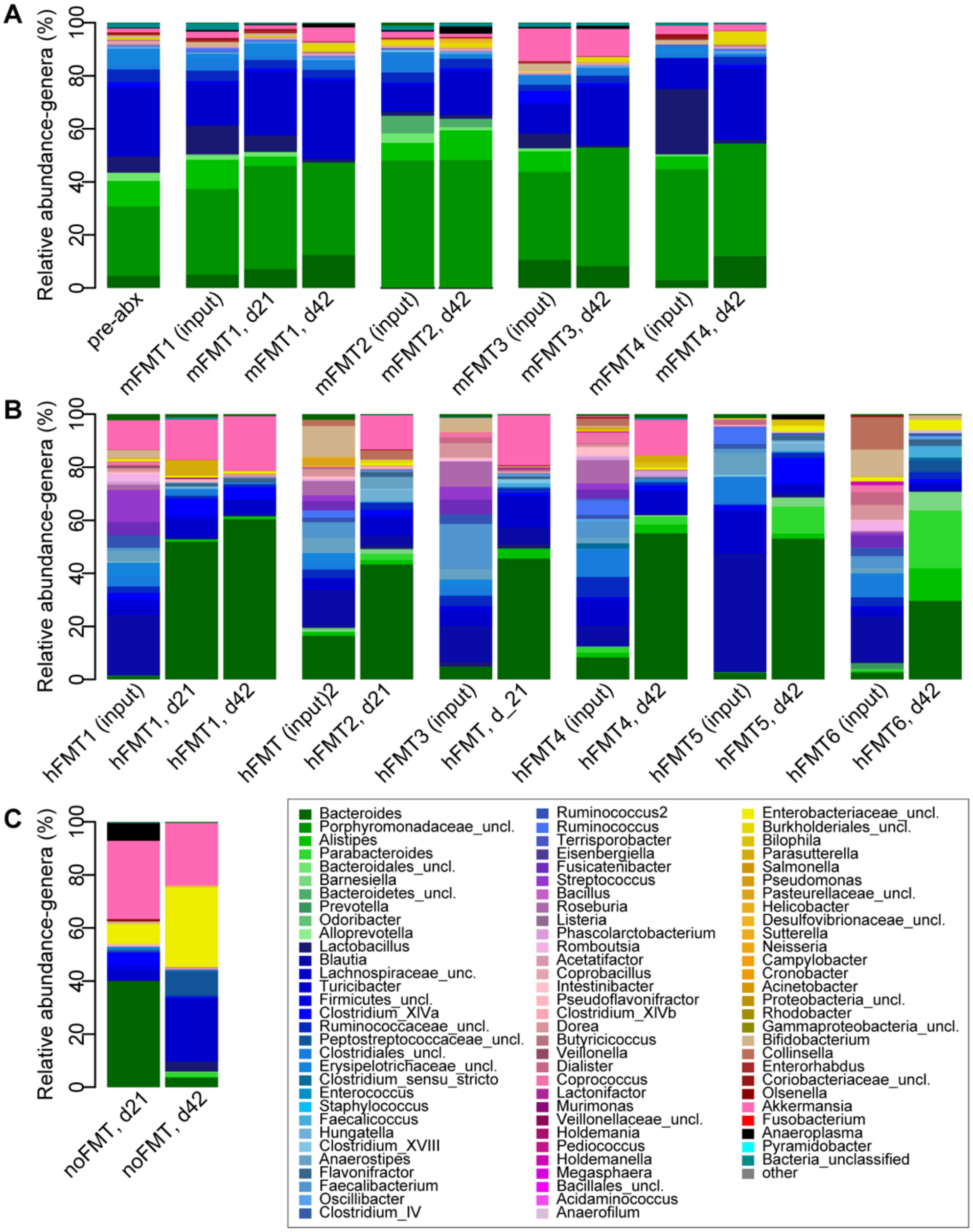

Figure S1. Genus-level taxonomy across mice by donor type. Average relative abundance of top 98% genera observed in FMT inputs and cecal samples in mice **A)** prior to any treatment (pre-abx) or after different individual mFMT sources, **B)** different individual hFMT sources, or **C)** no FMT, at indicated timepoints (day 21 or 42 post-infection).

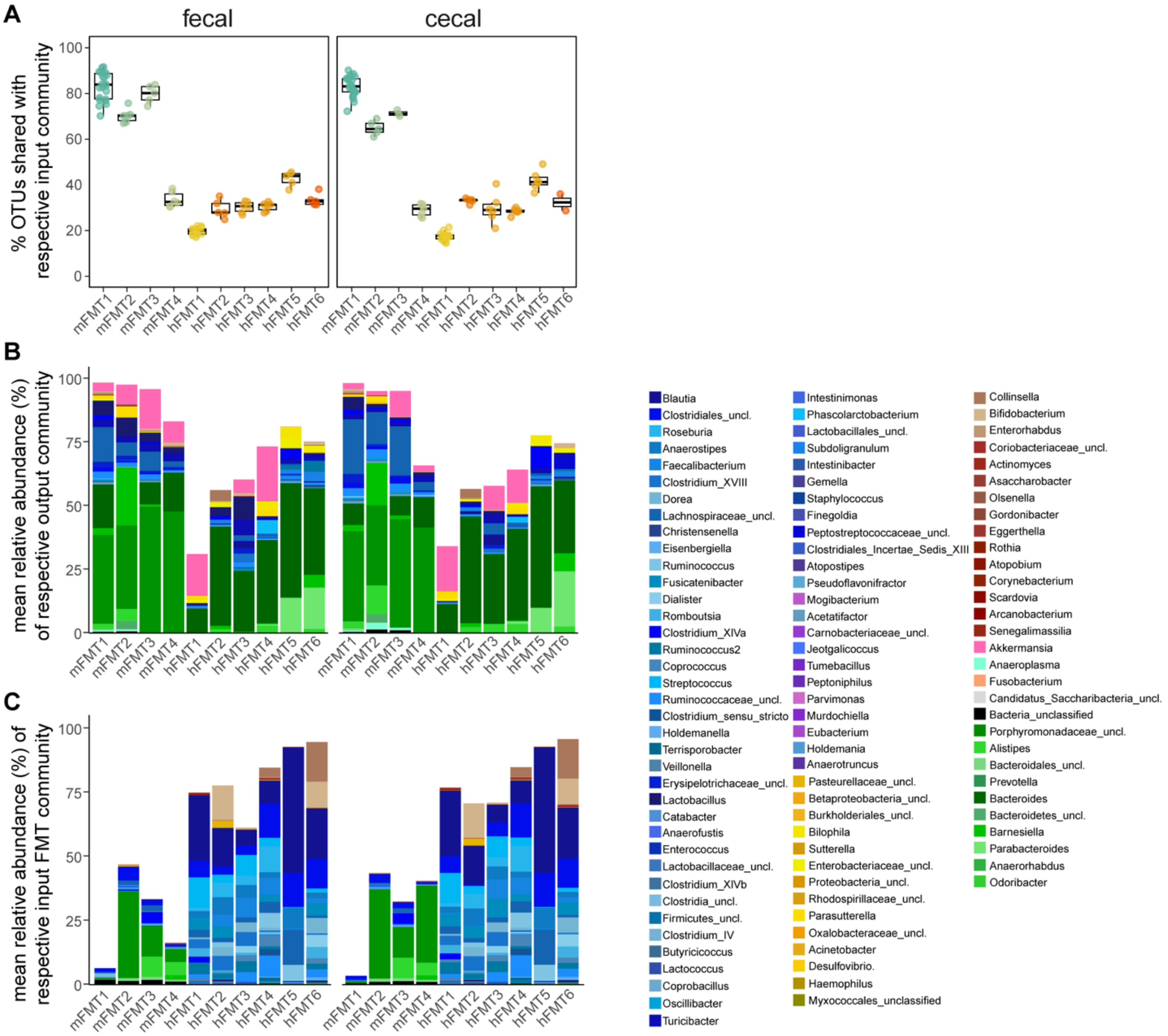

Figure S2. OTU engraftment across mice by donor type. Percent of OTUs (based on 16S rRNA gene-based counts) engrafting from mFMT vs hFMT by **A)** presence/absence of individual OTUs or **B)** relative abundance explained by engrafted OTUs in fecal or cecal samples, versus **C)** relative abundance of OTUs from each FMT input that did not engraft.

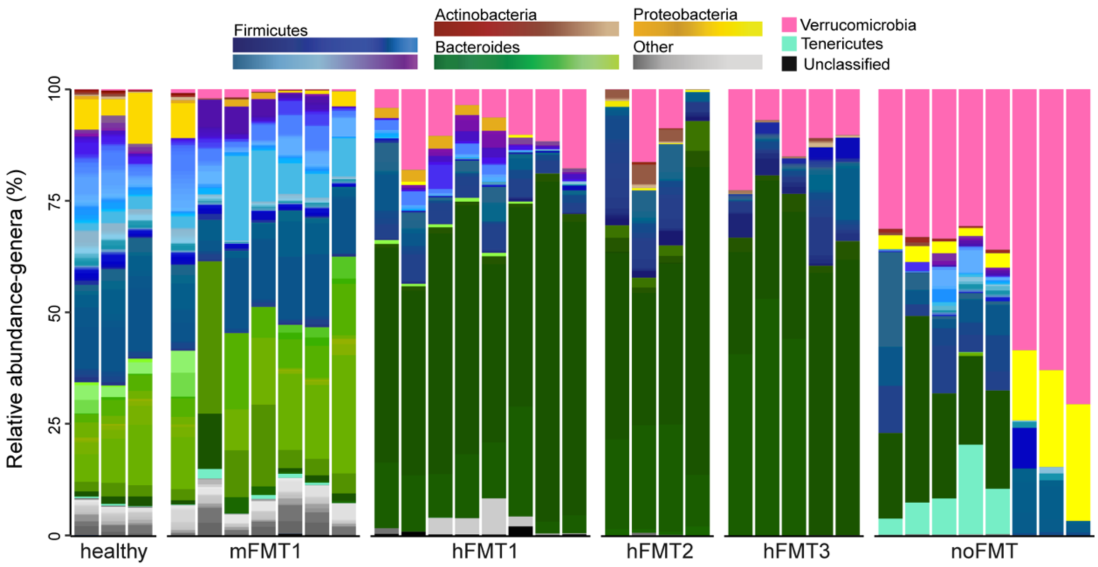

Figure S3. Relative abundance of individual mice. Relative abundance of genera identified by MetaPhlAn4 from cecal samples of mice. Each bar represents an individual mouse.

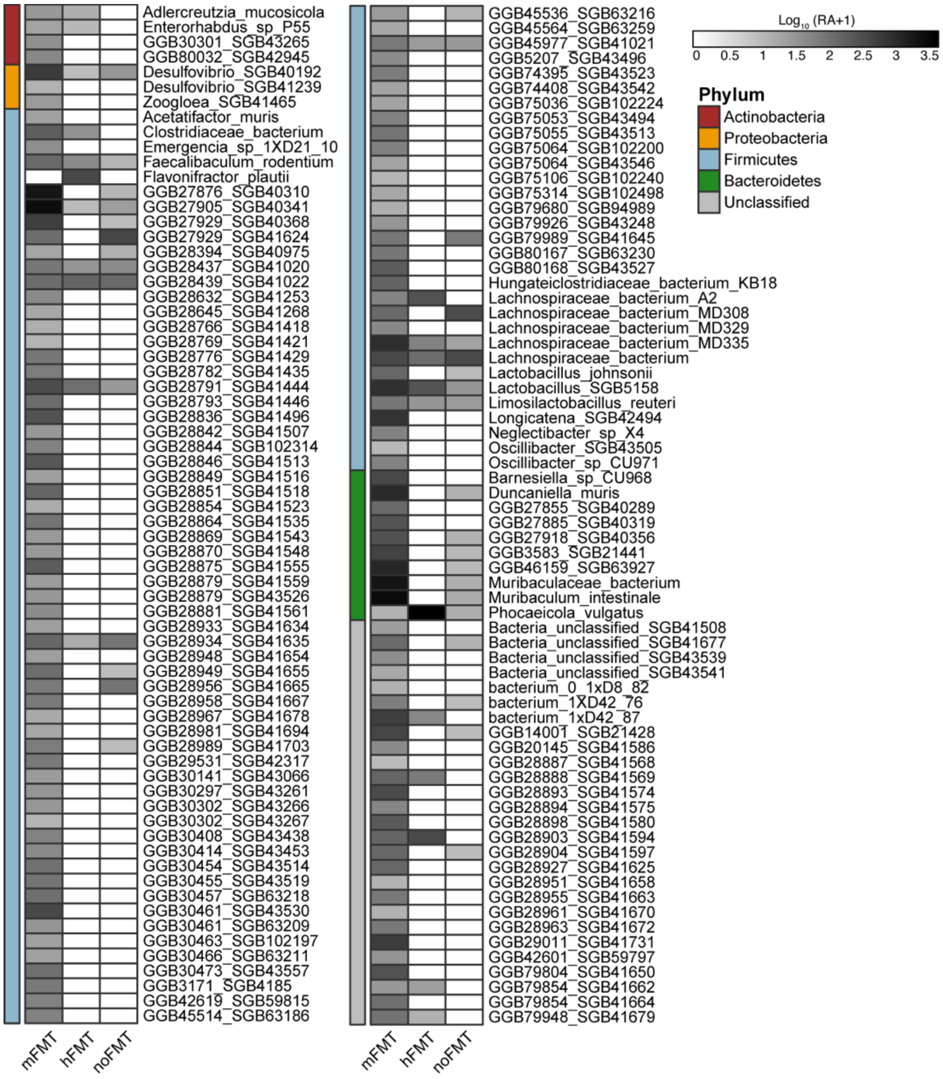

Figure S4. Bacterial genera significantly different in abundance between cleared and colonized mice. Mean $\log_{10}$-transformed (CPM + 1) of all bacterial genera significantly different in abundance between mice that cleared (mFMT) or did not clear (hFMT, noFMT) *C. difficile* (based on MaAsLin2; linear model with BH correction, $q \leq 0.01$).

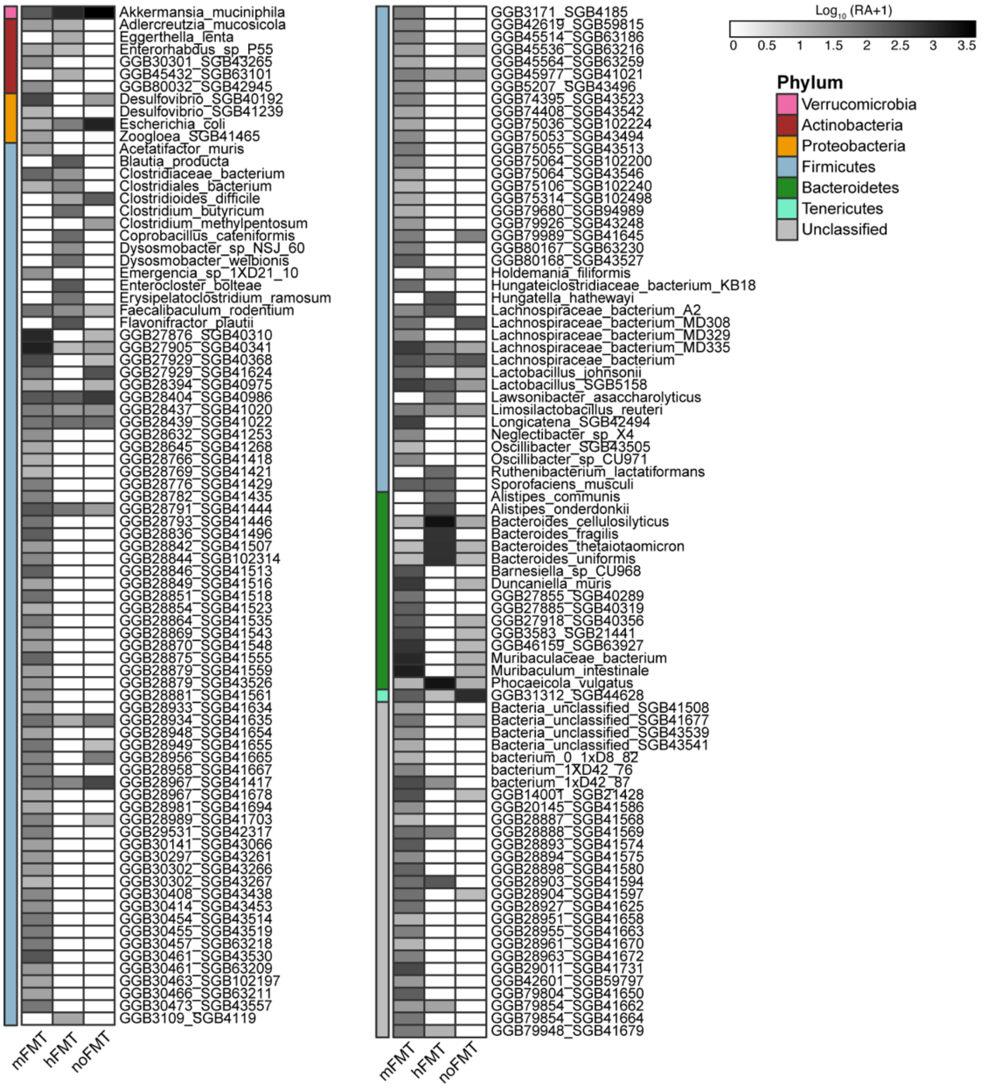

Figure S5. Bacterial genera significantly different in abundance across treatment groups. Mean log_{10}-transformed (CPM + 1) of all bacterial genera significantly different in abundance across treatment groups compared to mice treated with mFMT (based on MaAsLin2; linear model with BH correction, $q \leq 0.01$).

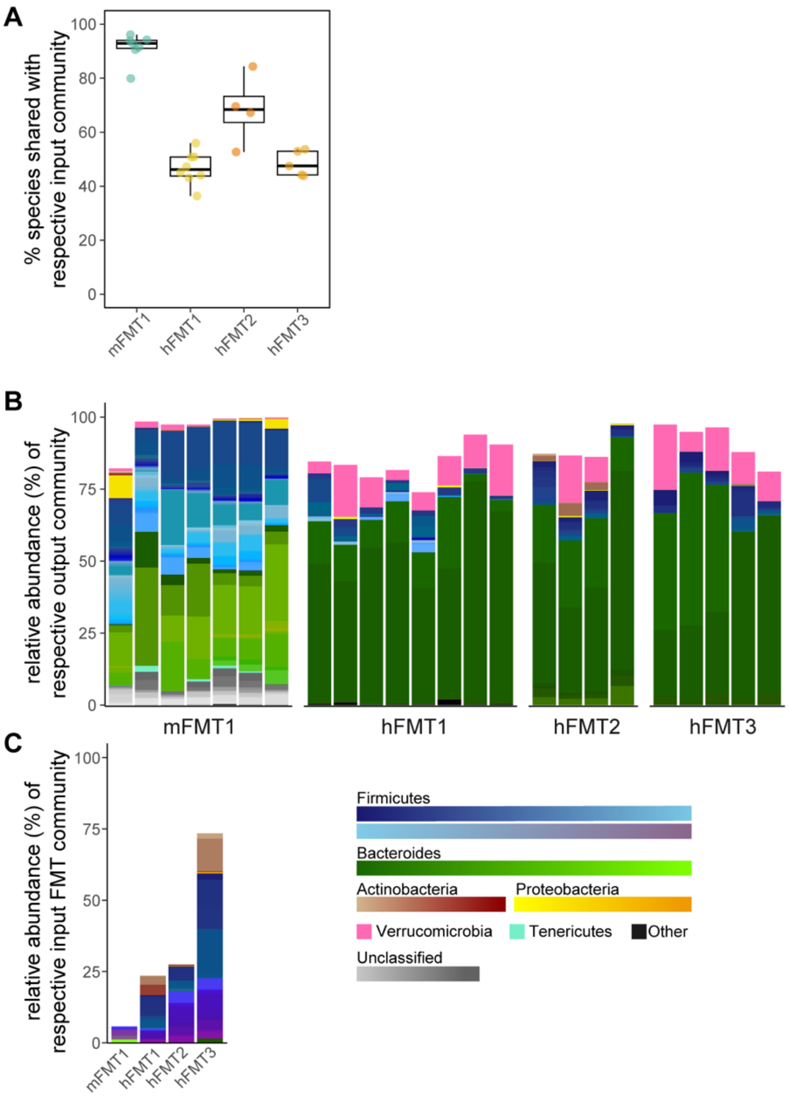

Figure S6. Species engraftment across mice by donor type. Percent of species (based on metagenomic taxonomy) engrafting from mFMT vs hFMT by **A)** presence/absence of individual species or **B)** relative abundance explained by engrafted species in cecal samples, versus **C)** relative abundance of species from each FMT input that did not engraft.

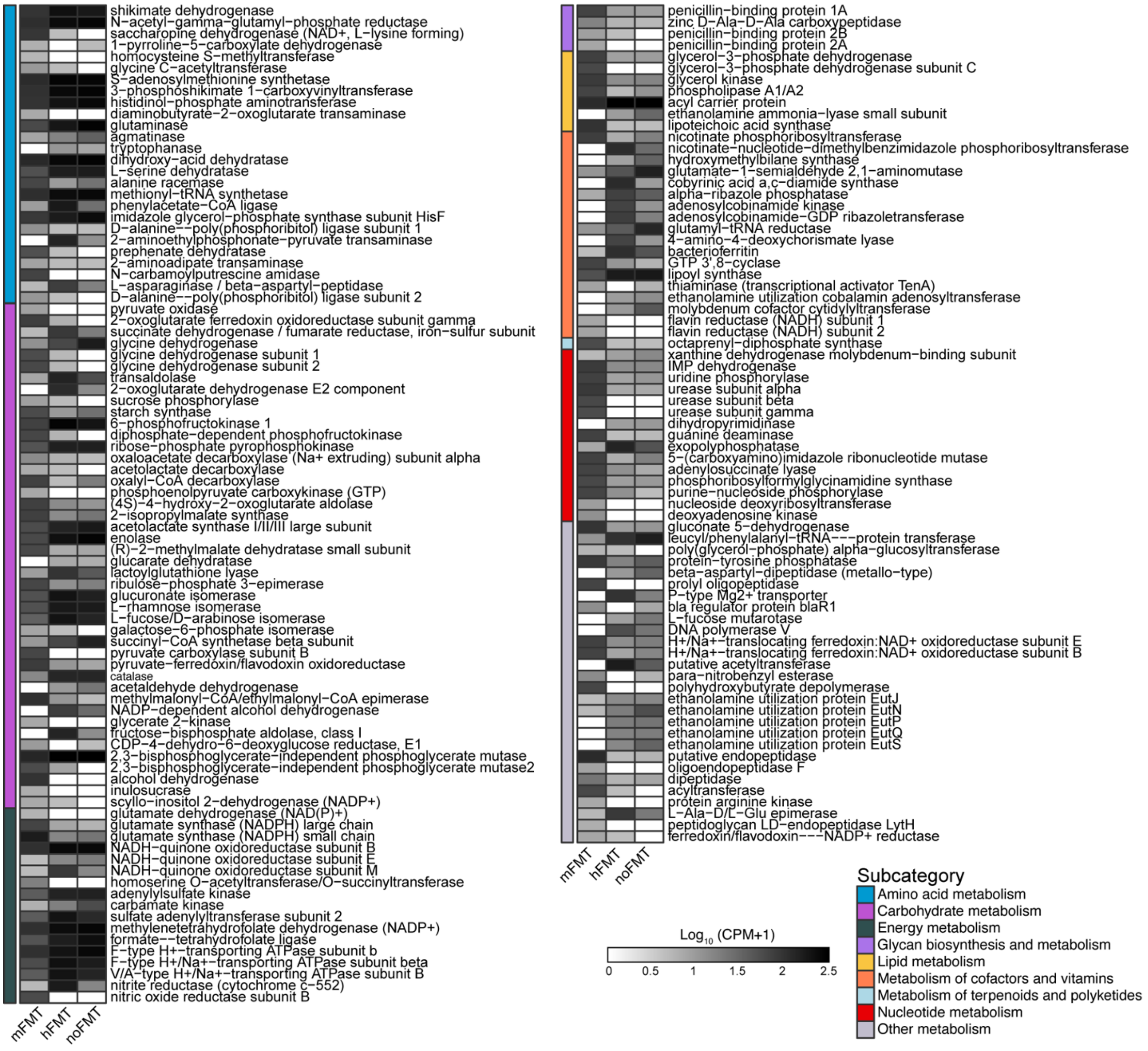

Figure S7. Microbial genes (KOs) associated with metabolism significantly different in abundance between cleared and colonized mice. Mean $\log_{10}$-transformed (CPM + 1) of metabolism associated KEGG orthologs (KOs) significantly different in abundance between mice that cleared (mFMT) or did not clear (hFMT, noFMT) *C. difficile* (based on MaAsLin2; linear model with BH correction, $q \leq 0.001$).

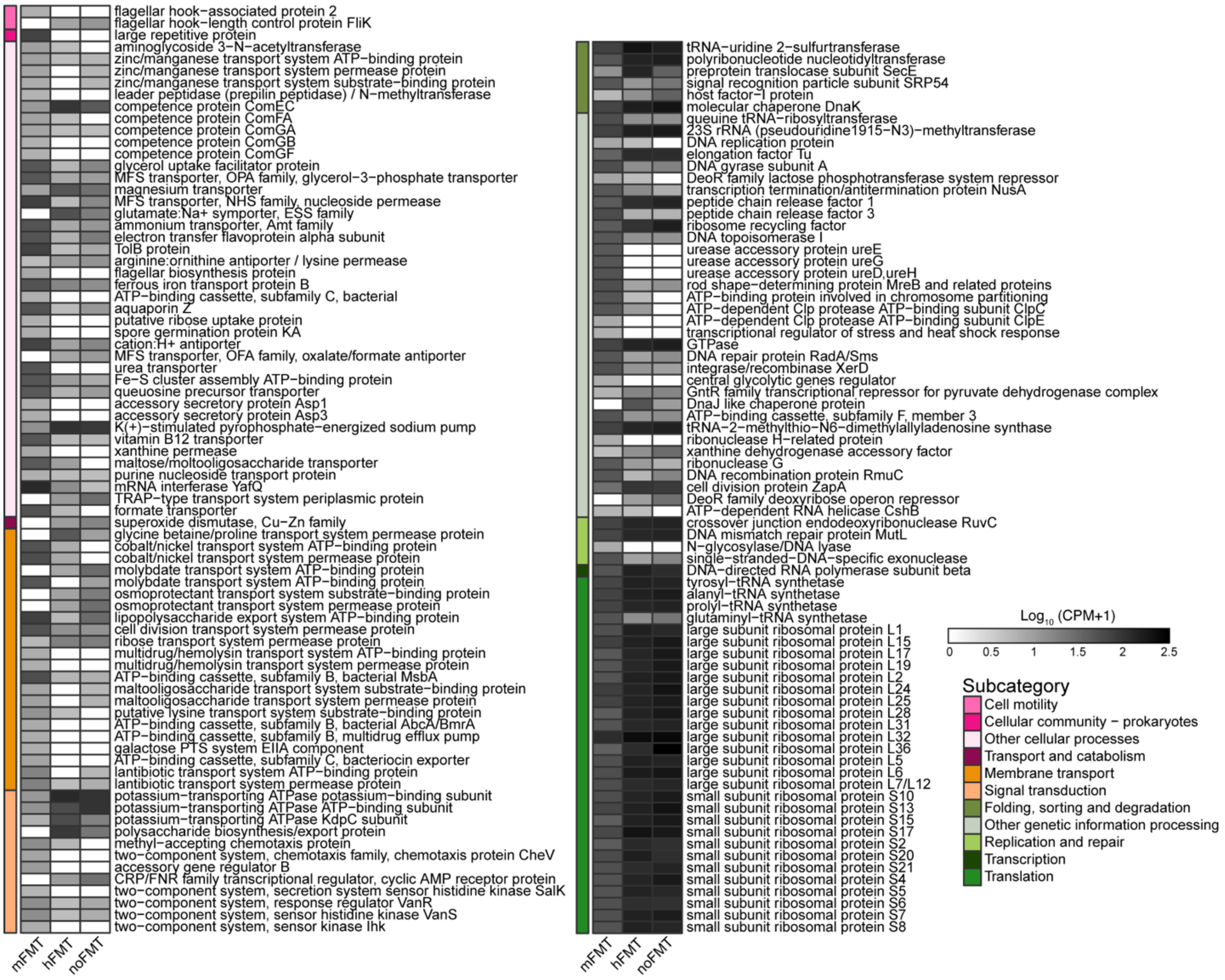

Figure S8. Microbial genes (KOs) not associated with metabolism significantly different in abundance between cleared and colonized mice. Mean $\log_{10}$-transformed (CPM + 1) of KOs from non-metabolism categories significantly different in abundance between mice that cleared (mFMT) or did not clear (hFMT, noFMT) *C. difficile* (based on MaAsLin2; linear model with BH correction, $q \leq 0.001$).

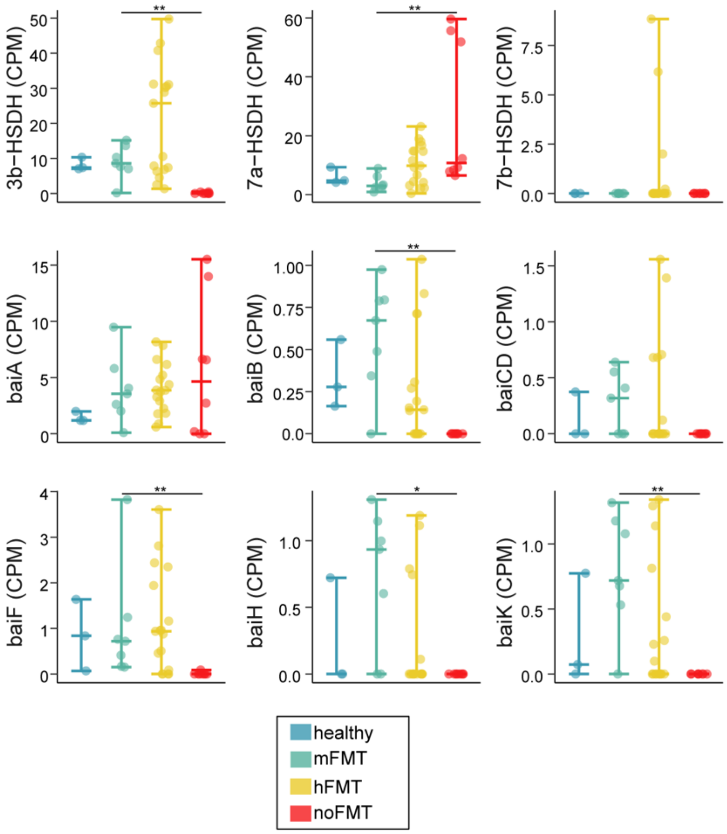

Figure S9. Abundance of microbial genes associated with bai operon. CPM of Uniref90 genes associated with the bile acid inducible (bai) operon. Statistical significance determined using Kruskal-Wallis test, with a post-hoc Dunn test (*$p < 0.01$, **$p < 0.001$).

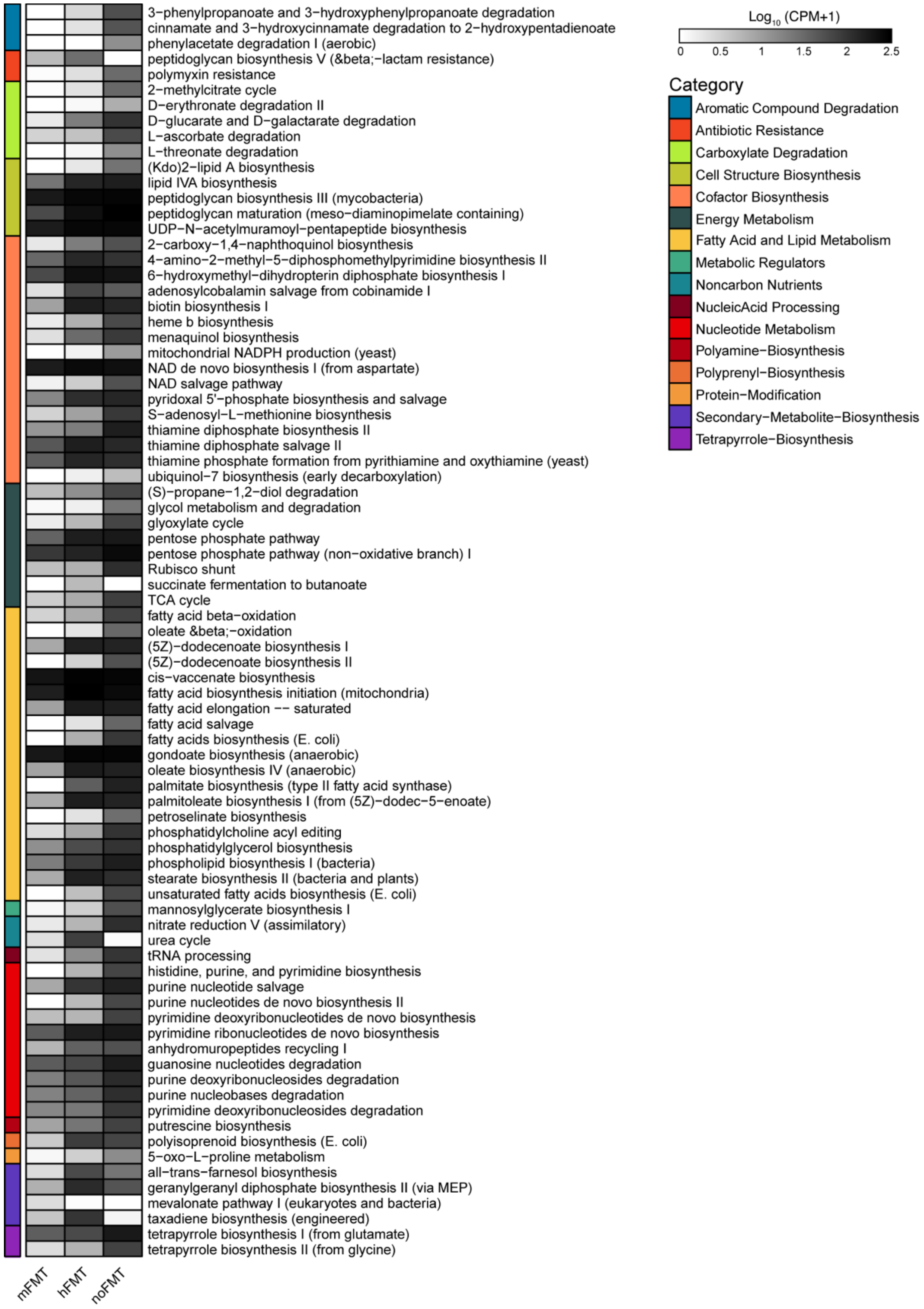

Figure S10. Microbial pathways associated with other metabolism significantly different in abundance across treatment groups. Mean log10-transformed (CPM + 1) of other metabolism

MetaCyc Pathways significantly different across treatment groups compared to mice treated with mFMT (based on MaAsLin2; linear model with BH correction, $q \leq 0.001$).

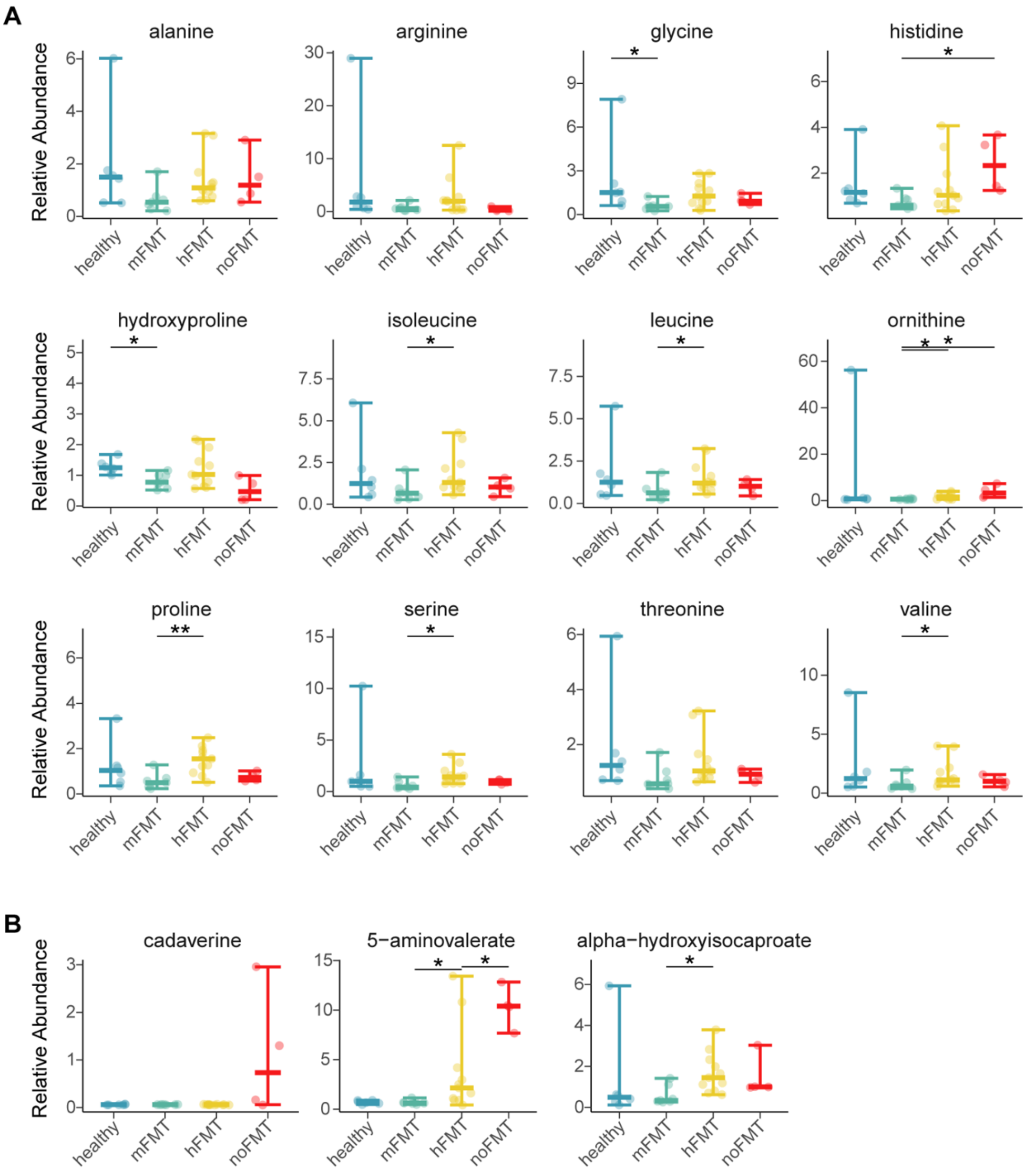

Figure S11. Abundance of Stickland metabolites across treatment groups. Median-scaled, minimum-imputed abundances of metabolites serving as **A)** inputs or **B)** outputs of Stickland metabolism. Statistical significance determined using Kruskal-Wallis test, with a post-hoc Dunn test (*$p < 0.01$, **$p < 0.001$).

Supplemental Methods

FMT inoculum preparation

Human FMT (hFMT) inocula were prepped in an anaerobic chamber (Coy Laboratories), adding 20 – 30 mg of frozen fecal material to 1.2 ml pre-reduced PBS in a 2-ml screw-top tube. The slurry was vortexed until homogenized, then allowed to gravity filtrate for at least 30 minutes to allow particles to settle before inoculation. Mouse FMT (mFMT) was prepped similarly, in that 5-7 fecal pellets from healthy, untreated mice were collected directly into a 2-ml screw-top tube that contained 1.2 ml pre-reduced PBS, vortexed intermittently until well-homogenized, then allowed to gravity filtrate prior to inoculation. A clear, disposable plastic gavage needle (Cadence Science, #9931) was used to deliver up to 200 µl of fecal slurry supernatant to each mouse via oral gavage, avoiding chunks that might clog the needle.

Preparation of the mFMT-spore inocula was adapted from Atarashi et al (1). 12 – 16 mouse fecal pellets were collected into 2.5 ml PBS, homogenized well, then placed at −80°C overnight. Fecal slurry was thawed, centrifuged at 1000 rpm for 30 seconds, and filtered using a 0.8 µM syringe filter (MilliporeSigma, #SLAAR33SB**).** The filtrate was vortexed at high speed for 20 minutes, heated at 50°C for 20 minutes, then vortexed again at medium speed for ~2 hour (all aerobically). The sample was stored at 4°C until inoculation the next day. 100 µl of spore preparation was delivered to each mouse via oral gavage.

Untargeted Metabolomics

Untargeted metabolomics from cecal content (50 – 100 mg; stored at −80°C until processing) was conducted by Metabolon (Durham, NC) using standard company procedures as described previously (2). Briefly and as per company platform, an automated MicroLab STAR system (Hamilton Company, Reno, NV) was used for sample preparation, which included precipitating protein with methanol under vigorous shaking and centrifugation. The extract was then aliquoted into five fractions for four methods total, described below. Organic solvent was removed using a TurboVap (Zymark, Hopkinton, MA) and stored (dried) under nitrogen overnight. Several control samples were prepared in parallel to samples, including: a pooled sample from all experimental samples, water blanks, and a cocktail of standards spiked into each sample. Median relative standard deviation for standards spiked into each sample was used to account for instrument variability. A Waters ACQUITY ultra-performance liquid chromatography (UPLC) and a Thermo Scientific Q-Exactive high resolution/accurate mass spectrometer interfaced with a heated electrospray ionization (HESI-II) source and Orbitrap mass analyzer operated at 35,000 mass resolution was used for all four methods, using the following solvents: 1) an aliquot chromatographically optimized for more hydrophilic compounds was gradient eluted from a C18 column (Waters UPLC BEH C18-2.1x100 mm, 1.7 µm) using water and

methanol, containing 0.05% perfluoropentanoic acid (PFPA) and 0.1% formic acid (FA) for reverse phase (RP)/UPLC-MS/MS with positive ion mode electrospray ionization (ESI); 2) an aliquot for the same method, but chromatographically optimized for more hydrophobic compounds, gradient eluted on the same C18 column but using methanol, acetonitrile, water, 0.05% PFPA, 0.01% FA, and operated at an overall higher organic content; 3) an aliquot analyzed using basic negative ion optimized conditions using a separate dedicated C18 column, gradient eluted from the column using methanol and water with 6.5mM Ammonium Bicarbonate at pH 8; and 4) an aliquot for analysis via negative ionization, eluted from a HILIC column (Waters UPLC BEH Amide 2.1x150 mm, 1.7 µm) with a gradient consisting of water and acetonitrile with 10mM Ammonium Formate, pH 10.8. The MS analysis alternated between MS and data-dependent MS^n scans using dynamic exclusion. The scan range varied slighted between methods but covered 70-1000 m/z.

Metabolon's hardware and software was used to extract data, identify peaks, and conduct data QC. A Metabolon-generated library of > 3,300 purified standards or recurrent unknown entities was used for compound identification, which includes authenticated standards with retention time/index (RI), mass to charge ratio (*m/z)*, and chromatographic data (including MS/MS spectral data). Identification was confirmed via three criteria: retention index within a narrow RI window of the proposed identification, accurate mass match to the library +/- 10 ppm, and the MS/MS forward and reverse scores (based on comparison of ions in experimental spectrum and the library spectrum) between the experimental data and authentic standards. Proprietary visualization and interpretation software developed by Metabolon was used to curate high quality data. Metabolites were quantified and normalized using area-under-the-curve, using a data normalization step tp correct for instrument variation over time. Each compound was corrected to a median equal to one and normalizing each data point proportionally ("block correction"). The 'ScaledImpData' resulting from these data was used for analysis in the current study, detailed in the scripts located in the github page: https://github.com/SeekatzLab/mouseCDI-SPF-hFMT.

Targeted Metabolomics

Cecal bile acid and SCFA quantification was conducted by the University of Michigan Metabolomics Core. For bile acids, a two-phase extraction was conducted on ~25 mg of cecal material. First, 1000 µl of chilled ethanol with isotope-labeled internal standard was added, and sonicated at 40% power, 20% duty cycle for 5 minutes, then incubated on ice for 10 minutes before remixing. 250 µl of this sample was added to a 12x75 mm glass tube, centrifuged, and split into supernatant (transferred to a microtube) and remnant in glass tube. Next, 200 µl of chilled methanol:chloroform 1:1 mix was added to the glass tube, vortexed, and incubated on ice for 10 minutes. After centrifuging this sample, it was combined with the ethanol-extracted supernatant and dried using a speedvac set to 45°C for ~45 minutes. The dried sample was

reconstituted with 1:1 methanol:water, removing 10 µl from each sample for a pooled sample for QC. 100 µl of sample was ran for LC-MS analysis using an Agilent system consisting of 1290 UPLC module coupled with a 6490 Triple Quad (QqQ) mass spectrometer (Agilent Technologies, Santa Clara, CA) operated in MRM mode, using MRM transitions as described previously (3). Metabolites were separated on a 100mm x 2.1mm Acquity BEH UPLC (1.7 uM) column (Waters Corp, Milford, MA) using H_2O / 0.1% Formic acid as mobile phase A, and Acetonitrile / 0.1% Formic acid as mobile phase B. The flow rate was 0.25 mL/min with the following gradient: linear from 5 to 25% B over 2 minutes, linear from 25 to 40% B over 14 mins, linear from 40 to 95% B over 2 minutes, followed by isocratic elution at 95% B for 5 minutes. The system was returned to starting conditions (5% B) in 0.1 min and held there for 3 minutes to allow for column re-equilibration before injecting another sample. The mass spectrometer was operated in ESI- mode according to previously published conditions.

For cecal SCFA analysis, ~50mg of material was combined with 400 uL of 30 mM hydrochloric acid containing isotopically-labeled acetate (0.125 mM), butyrate (0.125mM), and hexanoate (0.0125mM). Samples were sonicated for 20 seconds (Branson 450 Sonifier, Brookfield, CT) at power level 4, duty cycle 40%. 250 µl of methyl tert-butyl ether (MTBE) was added to each sample and vortexed for 10 seconds to emulsify, held at 4°C for 5 minutes, then vortexed again for 10 seconds. Solvent layers were separated by centrifugation for 1 minute. 10 µl of the MTBE layer was removed from each sample and pooled for QC purposes, before loading the MTBE sample layer into an autosampler. GC-MS on the sample was performed on an Agilent 69890N GC -5973 MS detector using a 1µL injection with a 1:10 split ratio on a ZB-WAXplus, 30m x0.25mmx0.25um (Phenomenex Cat#7HG-G013-11) GC column, with He as the carrier gas at a flow rate of 1.1ml/min. The injector and column temperatures were 240°C and 310°C (isocratic). Calibration standards were prepared along with samples to quantify metabolites.

Data for both cecal bile acids and SCFAs were processed with MassHunter Quantitative analysis version B.07.00, normalizing each metabolite to the nearest isotope-labeled standard and quantified using two replicated injections of seven standards to calibrate a linear curve (> 80% for each standard). Data were normalized to the wet cecal weight.

For SCFA measurement from fecal samples, 200 µl of diluted fecal slurry (1:10 in PBS) was added to a 0.22µm 96-well filter plate (MilliporeSigma, #MSGVS2210) and centrifuged at 4500 x g for 15 min at 4°C. Screw cap vials (Leap PAL Parts, #Vial-1.5-ND9-CG-100, #XAR-1521) with 100 µl inserts (Leap PAL Parts, #XQS1015) were loaded with fecal filtrate for analysis by high-performance liquid chromatography (Shimadzu Scientific Instruments, Columbia, MD) at the UMICH Microbiome Core using previously described instrumentation and protocol (4). Briefly, a flow rate of 0.6 ml per min, mobile phase of 0.01 N H_2SO_4, and column oven temperature of 50°C was used to run 10 µl injections of filtrate over an elution time of 40 minutes. Standard preparations of acetate, propionate, and butyrate at 0.1, 0.25, 0.5, 2.5, 5 20 and

20 mM were ran before and after each 96-sample preparation for peak identification and determination of compound concentration, with samples organized randomly. Baseline chromatograph corrections were conducted initially using the Shimadzu LC Software, assessing quality of peaks by relative retention time and peak width. Standard normal curves based on standards ran with each sample set were used to calculate concentrations, normalizing this to wet weight of fecal material (mmol/kg). Peaks outside of peak width cutoffs for the relative retention time were rejected and not included in analysis.

References

1. Atarashi K, Tanoue T, Oshima K, Suda W, Nagano Y, Nishikawa H, Fukuda S, Saito T, Narushima S, Hase K, Kim S, Fritz J V., Wilmes P, Ueha S, Matsushima K, Ohno H, Olle B, Sakaguchi S, Taniguchi T, Morita H, Hattori M, Honda K. 2013. Treg induction by a rationally selected mixture of Clostridia strains from the human microbiota. Nature 500:232–236.

2. McMillan AS, Zhang G, Dougherty MK, McGill SK, Gulati AS, Baker ES, Theriot CM. 2024. Metagenomic, metabolomic, and lipidomic shifts associated with fecal microbiota transplantation for recurrent *Clostridioides difficile* infection. mSphere 9.

3. Griffiths WJ, Sjövall J. 2010. Bile acids: Analysis in biological fluids and tissues. J Lipid Res https://doi.org/10.1194/jlr.R001941-JLR200.

4. Baxter NT, Schmidt AW, Venkataraman A, Kim KS, Waldron C, Schmidt TM. 2019. Dynamics of Human Gut Microbiota and Short-Chain Fatty Acids in Response to Dietary Interventions with Three Fermentable Fibers.
